# Foot-Worn Inertial Sensors Are Reliable to Assess Spatiotemporal Gait Parameters in Axial Spondyloarthritis under Single and Dual Task Walking in Axial Spondyloarthritis

**DOI:** 10.3390/s20226453

**Published:** 2020-11-12

**Authors:** Julie Soulard, Jacques Vaillant, Romain Balaguier, Athan Baillet, Philippe Gaudin, Nicolas Vuillerme

**Affiliations:** 1 University Grenoble Alpes, AGEIS, 38000 Grenoble, France; jacques.vaillant@gmail.com (J.V.); Romain.balaguier@hotmail.fr (R.B.); nicolas.vuillerme@univ-grenoble-alpes.fr (N.V.); 2 CHU Grenoble Alpes, 38000 Grenoble, France; 3 University Grenoble Alpes, CNRS, CHU Grenoble Alpes, Grenoble INP, TIMC-IMAG UMR5525, 38000 Grenoble, France; abaillet@chu-grenoble.fr (A.B.); pgaudin@chu-grenoble.fr (P.G.); 4 Institut Universitaire de France, 75000 Paris, France; 5 LabCom Telecom4Health, University Grenoble Alpes & Orange Labs, 38000 Grenoble, France

**Keywords:** ankylosing spondylitis, spondylarthritis, walking, wearable sensors, 10-m walk test, reliability, manual task, dual-task

## Abstract

The aim of this study was (1) to evaluate the relative and absolute reliability of gait parameters during walking in single- and dual-task conditions in patients with axial spondyloarthritis (axSpA), (2) to evaluate the absolute and relative reliability of dual task effects (DTE) parameters, and (3) to determine the number of trials required to ensure reliable gait assessment, in patients with axSpA. Twenty patients with axSpa performed a 10-m walk test in single- and dual-task conditions, three times for each condition. Spatiotemporal, symmetry, and DTE gait parameters were calculated from foot-worn inertial sensors. The relative reliability (intraclass correlation coefficients-ICC) and absolute reliability (standard error of measurement-SEM and minimum detectable change-MDC) were calculated for these parameters in each condition. Spatiotemporal gait parameters showed good to excellent reliability in both conditions (0.59 < ICC < 0.90). The reliability of symmetry and DTE parameters was low. ICC, SEM, and MDC were better when using the mean of the second and the third trials. Spatiotemporal gait parameters obtained from foot-worn inertial sensors assessed in patients with axSpA in single- and dual-task conditions are reliable. However, symmetry and DTE parameters seem less reliable and need to be interpreted with caution. Finally, better reliability of gait parameters was found when using the mean of the 2nd and the 3rd trials.

## 1. Introduction

Gait is a fundamental human motor activity [1] and allows for movement from place to place in daily life, and thus social participation [2]. Assessing gait does offer undeniable prospects in a clinical setting. For instance, slow gait speed (i.e., <1.0 m/s) is indeed associated to three times greater limitations in social participation [2]. Furthermore, gait outcomes can be used to quantify therapeutic interventions [3], or predict diverse outcomes including falls [4], cognitive decline [5], or mortality [6].

To assess gait performance, wearable inertial sensors are now affordable, transportable, and easy to use [7]. These devices advantageously allow for the computation of spatiotemporal gait parameters and could help to better evaluate gait characteristics in clinical practice [7]. Wearable inertial sensors can be placed, for instance, on the trunk, shank, or feet to perform gait assessment in a corridor, using devices easily transportable between care units. Wearable sensors allow for the analysis of multiple gait cycles in longer distances than laboratory-based gait assessments, which could even be performed continuously in a patients’ home in an ecological environment. Moreover, to better adapt gait test to the motor-cognitive challenges that occur during every-day ambulation, dual-tasking paradigms are widely used in the clinical setting [8]. This consists of “the concurrent performance of two tasks that can be performed independently and have distinct and separate goals” [9] and was considered as closer to daily-living walking [10]. The use of gait dual-tasking paradigm has been shown to allow better classification and diagnosis in various populations such as patients with neurologic disorders (i.e., Parkinson disease [11], multiple sclerosis [12] or stroke [13]), with knee pain [14] or osteoarthritis [15], or older adults with cognitive impairment [16] or at risk of falls [17,18]. Dual task effects (DTE) assess the change in performance on the primary task (e.g., gait) while concurrently performing a secondary task (e.g., counting backward, carrying a full cup of water…) [19].

To separate disease or treatments effects to measurement errors of the system (i.e., inertial sensors), the reliability of these devices has to be evaluated [20]. More precisely, the intra-session reliability refers to the reliability of different trials performed during the same session [21,22]. It is recommended to better describe the sources and magnitudes of errors in gait analysis [21,23] and to compare performances between groups at a single time point [24]. Intra-session reliability can favorably be used to assess the number of trials to ensure reliable measurements [25,26,27,28] to further optimize gait assessments in clinical practice. The relative reliability (i.e., intraclass correlation coefficients (ICC)) and the absolute reliability (i.e., standard error of measurement (SEM) and minimum detectable change (MDC)) have complementary to be reported and are crucial in rehabilitation to interpret changing scores in patients [29]. Axial spondyloarthritis (axSpA) is a chronic rheumatic disease, which affects the vertebral column and sacroiliac joints [30]. This disease can lead to postural changes such as loss of lumbar lordosis, exaggeration of thoracic kyphosis, and difficulty in looking upwards [31]. Static and dynamic postural changes [32,33,34] and foot abnormalities [35] can also be associated, and thus have consequences on spatiotemporal and kinematic gait parameters, i.e., gait performance [36]. Previous studies [37,38,39,40] reported decreased lower limb angles in the sagittal plane and decreased pelvic tilt associated to lower variability among patients with axSpA compared to healthy controls [41]. However, to the best of our knowledge, no study has assessed the reliability of gait assessments in this population.

Thus, the aim of this study was to evaluate the intra-session reliability of gait assessment with foot-worn wearable inertial sensors, in single- and dual-task conditions, in patients with axSpA. More precisely, the aims were (1) to evaluate the relative and absolute reliability of gait parameters during walking in single- and dual-task conditions in patients with axSpA, (2) to evaluate the absolute and relative reliability of DTE parameters, and (3) to determine the number of trials required to ensure reliable gait assessment, in patients with axSpA.

## 2. Materials and Methods

### 2.1. Participants

Inclusion criteria were previously described [42]: (i) age between 18 to 65 years, (ii) diagnosis of axSpA according to ASAS/EULAR criteria [43] or ankylosing spondylitis according to Van der Linden et al. [44], (iii) ability to walk 180 m without technical help, and (iv) stable treatment for at least 12 months. 

All participants signed informed consent and approved to participate in the FOLOMI study (Function, Locomotion, Measurement and Inflammation), a prospective longitudinal study [42]. The study was approved by local ethic committee (CPP Ile De France 1, RCB: 2017-A03468-45, date of agreement: 17 July 2018, Last version: V6.0, 17 June 2020) and is registered in Clinical trials (NCT03761212). The present study respects the GRRAS guidelines regarding psychometric studies [45]. 

The sample size was calculated using sample size calculator [46,47] with a minimum acceptable reliability of 0.6, expected reliability of 0.85, significance level of 0.05, power of 80%, and 3 repetitions per subject, giving a sample size of 18.

### 2.2. Experimental Protocol

Patients’ characteristics (age, weight, height, body mass index) and disease characteristics (disease duration, pain intensity at time of evaluation assessed with visual analogue scale, BASDAI, BASFI, and morning stiffness) were collected.

Gait was assessed at least 2 hours from the end of morning stiffness of the patients as morning stiffness can have consequences on functional limitations [48]. A 10-meter walk test (10 MWT) was performed on a 14-m walkway, at comfortable walking speed [49], in single- and dual-task conditions (3 trials per condition). In the dual-task condition, participants had to carry a full cup of water in their dominant hand with the following instruction: “perform both tasks as well as possible” [50,51]. The examiner noted whether there was any spillage of water [51]. Gait assessments were performed by the same examiner (JS). Participants wore walking shoes, with 2 inertial measurement units with tri-axial accelerometers and gyroscopes (Physilog5^®^, 200 Hz, BioAGM, Gait Up, CH), placed above both feet (behind the base of the fifth metatarsal) [52]. 

### 2.3. Spatiotemporal Gait Parameters

Spatiotemporal gait parameters were calculated with the Gait Analysis Software (V5.3.0) provided by Gait Up (CH) [53]. The two first and last steps were removed from the analysis [54,55] and at least 16 steps were included in the analysis [56]. After checking for non-significant differences between spatiotemporal gait parameters of left and right feet, the mean of left and right feet for each parameter was calculated:

- Speed (m.s^−1^): Mean walking stride velocity of forward walking.
- Cadence (step/minute): Number of steps in a minute.
- Stride length (m): Distance between two consecutive footprints on the ground, from the heel of a foot to the heel of the same foot, one cycle after.
- Swing (%): Portion of the cycle during which the foot is in the air and does not touch the ground.
- Stance (%): Portion of the cycle during which part of the foot touches the ground.
- Double support (%): Portion of the cycle where both feet touch the ground.
- Load (%): Portion of the stance between the heel strike and the foot being flat on the ground.
- Foot Flat (%): Portion of the stance where the foot is fully flat on the ground.
- Push (%): Portion of the stance between the foot being flat on the ground and the toe leaving the ground at take-off.

In order to remove the variability between subject associated with size [57], speed, cadence, and stride length were normalized to have non dimensional values using the formula proposed by Hof [58] and Pinzone et al. [57]:

$$Normalized\;speed\;\left(speed\;N\right)\;=\;\;\frac{Speed}{\sqrt{gl_{0}}}$$

where *g* denotes the acceleration of gravity (=9.81 m/s^2^) and *l*_0_ leg length.

$$Normalized\;cadence\;\left(cadence\;N\right)\;=\;\;Cadence*\sqrt{\frac{l_{0}}{g}}$$

where *g* is the acceleration of gravity and *l*_0_ leg length.

$$Normalized\;stride\;length\;\left(stride\;length\;N\right)\;=\;\;\frac{stride\;length}{l_{0}}$$

where *l*_0_ denotes leg length.

### 2.4. Symmetry Gait Parameters

The symmetry index (SI) is the most commonly used and cited parameters [59]. It assesses symmetry between left and right feet, with a value of 0% indicating full symmetry and a value of 100% indicating asymmetry. *SI* was calculated with the following formula for each of the spatiotemporal gait parameter (except double support) [59]:

$$Symmetry\;Index\;\left(SI\right)\;=\;\;\frac{\left|\;X_{L}-X_{R}\;\right|}{0.5\;\times \left(X_{L}+X_{R}\right)}\times 100\%$$

where *X_L_* is the value of each parameter of left foot and *X_R_* the value of each parameter of right foot.

The symmetry ratio (SR), described as easier to interpret and recommended on the basis of potential clinical utility in stroke patients [60] was also computed. A *SR* of 1 indicates full symmetry, while a *SR* of 2.0 on gait speed indicates that the right foot is twice faster than left foot. It was calculated with the following formula for each of the spatiotemporal gait parameter (except double support) [60]:

$$Symmetry\;ratio\;\left(SR\right)\;=\;\;\frac{X_{R}}{X_{L}}$$

where *X_L_* denotes the value of each spatiotemporal gait parameter of left foot and *X_R_* the value of each spatiotemporal gait parameter of right foot.

### 2.5. Dual Task Effects Parameters

To assess the influence of the addition of a secondary attention demanding task on gait, the dual task effect (DTE) was also calculated [9,51]. DTE is the relative measure of change in performance [61]. DTE calculated while walking and carrying a cup has previously been shown to be more reliable than when calculated while walking and performing a cognitive task [62]. DTE was calculated from mean parameters as proposed by [51]:

(1) DTE = single task performance−dual task performance

*DTE* has the same unit as the gait parameter that is used in the calculation. A negative value of *DTE* for gait speed, cadence, stride length, swing, and foot flat is associated to better performance in the dual-task, while a negative value for stance, double support, load, and push is associated to worse performance in the dual-task [51]. 

*DTE* can also be calculated as a percentage, being unit-less, and permitting comparison between parameters [51]:

(2) $$DTE\;\%=\;\frac{\mathrm{single}\;\mathrm{task}\;\mathrm{performance}-\mathrm{dual}\;\mathrm{task}\;\mathrm{performance}}{\mathrm{single}\;\mathrm{task}\;\mathrm{performance}}\times 100$$

The same interpretation as *DTE* can be done regarding positive and negative *DTE*% values.

### 2.6. Data Analysis

Spatiotemporal gait parameters and symmetry gait parameters in single- and dual-task conditions, and *DTE* and *DTE*% values were normally distributed (Shapiro–Wilk normality test). 

To estimate the magnitude of the systematic difference in gait parameters between trials 1-2, trials 1-3, trials 2-3, 95% confidence interval (CI) of the mean difference (*Mean_Diff_*) were used. *CI* was calculated with the following formula:

$$CI\;=Mean_{Diff}\;\pm \;tn-1\;\times \surd \left(\frac{SD_{Diff}}{n}\right)$$

where *n* corresponds to the number of participants and *tn* − 1 corresponds to the value of *t* distribution with *n* − 1 of freedom. 

A repeated measures analysis of variance (RM-ANOVA) with the number of trials used as within subject factor was performed on gait parameters, ICC and SEM values. When significant effect of the number of trials was found, a Tukey post-hoc test was used to compare differences between trials. ICC, SEM, and MDC were used to compute the relative and absolute reliability across the trials 1-2-3 in each condition (single- and dual-task). The relative reliability was evaluated by the calculation of a 2-way fixed ICC_2,1_ (for absolute agreement). ICC values inferior to 0 were considered as poor reliability, between 0.01 to 0.2 as slight, between 0.21–0.4 as fair, between 0.41–0.6 as moderate, 0.61–0.8 as substantial, and 0.81–1.00 as almost perfect [63]. SEM, with the same unit as gait parameters, corresponds to the absolute measure of the variability of the errors of measurements and informs on the precision of gait parameters of individual participants [64]. SEM was calculated with the following formula [65]:

(3) SEM = SD√(1−ICC)

where *SD* is the standard deviation of the parameters from all patients, and *ICC* the relative reliability. *MDC* is the minimum value for which a difference can be considered as “real”, and was generated with the formula:

(4) MDC = SEM×1.96×√2

*SEM*% and *MDC*% were also calculated as a percentage of mean for each parameter.

Bland and Altman plots of the differences between trials against their mean and limits of agreements (LOA) were used to assess the magnitude of disagreement between trials. A difference between trials outside the LOA can be considered as real change.

*ICC*, *SEM* (3), *MDC* (4), *SEM*%, and *MDC*% were calculated with the same formula for *DTE* (1) and *DTE*% (2) [66].

## 3. Results

Twenty patients with axSpA (10 first men and 10 first women included in FOLOMI study) were included in the present study. Patients were 44.9 (10.7) years old, 169.0 (8.7) cm height, 74.9 (15.1) body weight, with a disease duration of 11.2 (9.5) years. Pain intensity at the time of evaluation was 3.3 (2.5) and morning stiffness was about 32.5 (32.3) minutes. BASDAI was 3.3 (1.9) and BASFI was 3.1 (2.0).

### 3.1. Intra-Session Relative and Absolute Reliability of Spatiotemporal Gait Parameters

Table 1 and Table 2 show, respectively, the means and standard deviations of spatiotemporal gait parameters obtained in the single- and the dual-task conditions for each trial, the mean of the three trials, and *p*-values obtained from RM-ANOVA with differences between trials.

Table 3 and Table 4 show relative (ICC) and absolute (SEM% and MDC%) reliability of spatiotemporal gait parameters for each trial, respectively, in single and dual-task conditions.

All ICC values for spatiotemporal and normalized gait parameters were above 0.81 in single- and dual-task conditions, except for swing, stance, and double support percentage in single-task condition where ICC values were above 0.61 (Table 3 and Table 4). MDC% values were comprised between 2.19% and 22.95% for these parameters in single- and dual-task conditions. 

Symmetry ratio parameters reliability was low but was acceptable when using the 2nd and 3rd trials (0.40 < ICC < 0.83, 4.63 < MDC% < 25.06 in single-task; 0.00 < ICC < 0.79, 3.50 < MDC% < 20.17 in dual-task), while symmetry index parameters reliability was weaker (0.25 < ICC < 0.75, 121.08 < MDC% < 298.61 in single-task; 0.00 < ICC < 0.79, 86.38 < MDC% < 395.84 in dual-task). 

Supplementary Tables S1 and S2 present limits of agreement, SEM and MDC values for the spatiotemporal and symmetry parameters in single- and dual-task conditions.

Supplementary Figure S1 presents Bland and Altman plots for speed, cadence, and stride length parameters in single- and dual-task conditions. 

### 3.2. Intra-Session Relative and Absolute Reliability of Dual Task Effects Parameters 

Table 5 and Table 6 show respectively means and standard deviations of dual task effects and dual task effects percentages of spatiotemporal gait parameters for each trial, the mean of the 3 trials, and *p*-values obtained from RM-ANOVA with differences between trials.

Table 7 and Table 8 show reliability of DTE and DTE% for each spatiotemporal gait parameters using ICC, SEM, and MDC. 

ICC values for the speed, stride length DTE and DTE% were above 0.61.

ICC values for the cadence, load ratio DTE and DTE% were comprised between 0.41 and 0.8. 

ICC values for the foot flat and push ratios, and swing, stance, and double support percentage DTE and DTE% were under 0.6. 

Swing, stance, and double support percentages DTE% showed better reliability for trials 2-3 with ICC values comprised between 0.49 and 0.59. DTE% parameters showed better relative reliability (i.e., higher ICC) than DTE parameters. 

Supplementary Tables S3 and S4present limits of agreement, SEM, and MDC values for DTE and DTE% of spatiotemporal gait parameters.

Supplementary Figure S1 presents Bland and Altman plots for speed, cadence, and stride length DTE parameters.

### 3.3. Number of Trials to Ensure Reliable Measurements

The mean values of gait parameters obtained in the single- and dual-task conditions were not significantly different between trials whatever the conducted comparisons with *p*-values ranging from 0.73 to 1.0 (Table 1 and Table 2). Same results are observed with DTE and DTE% where no statistically differences were found. 

The comparisons of means of ICC between trials showed that slightly higher ICC and lower SEM and MDC were obtained when pooling gait parameters from the second and the third trials, compared to the first and second trials, to the first and third trials, or to the three trials in the single- and the dual-task conditions.

## 4. Discussion

The study demonstrated excellent intra-session reliability of spatiotemporal and normalized gait parameters assessed by foot-worn inertial sensors under single and dual-task walking in patients with axSpA. This study also gives SEM and MDC of basic spatiotemporal gait parameters, in single- and dual-task conditions, for this population. The reliability was better for the second and the third trials, suggesting that the first trial could be considered as a warming before real assessments. The present study demonstrated low reliability of symmetry gait parameters and dual task effects obtained from spatiotemporal gait parameters. 

### 4.1. Reliability of Gait Parameters in Patients with axSpA

Gait parameters measured in patients with axSpA were reliable in both single- and dual-task conditions. Although the population is different, this result is in line with previous reviews reporting good to excellent reliability of single and dual-task assessments in older adults [67] or patients with neurological conditions [68]. We showed that relative reliability of spatiotemporal and gait parameters obtained in single- and dual-task conditions, as assessed by ICC, was considered as excellent for most of the parameters (0.81 < ICC < 0.98). The swing (%), stance (%) and double support (%) times were slightly less reliable than the other spatiotemporal gait parameters, but showed still moderate to perfect reliability (0.59 < ICC < 0.90). Symmetry gait parameters showed low relative reliability (Table 5 and Table 6) in line with results reported from previous studies with healthy participants [3,69,70,71]. Symmetry ratio presented better reliability than symmetry index, notably for the second and third trials with ICC comprised between 0.40 and 0.83 considered as moderate to substantial reliability.

SEM and MDC were acceptable in single- and dual-task conditions. For spatiotemporal and symmetry ratio parameters, a change of 2–25% of performance (2.19 < MDC% < 25.06) could be identified but lower absolute reliability was found for symmetry index parameters (86.38 < MDC% < 395.84). These results are comparable to those reported in recent studies in which Physilog^®^ inertial sensors were also to measured spatiotemporal gait parameters [52,72]. Indeed, Lefeber et al. in 2019 reported a test retest reliability of gait parameters at self-selected comfortable speed on a 12-m walkway, with 20-min break between sessions in patients after a stroke using foot-worn sensors. Comparable results were reported with ICC values ranging from 0.546 to 0.985, with lower reliability for stance and swing phase percentage (0.547) [52]. Lower reliability of these parameters was explained by less apparent negative peaks in foot pitch angular velocity related to weakened foot lifters, leading to lower detection of heel strike and/or toe off. Standard error of measurement (SEM) observed in the present study (SEM values comprised from 0.01 to 1.93 in single task, Supplementary Table S1) were lower than those reported by Lefeber et al. in patients post stroke [52] with SEM values comprised between 0.038 and 4.344. Most of the higher values of SEM, obtained in Lefeber et al.study [52], were those of paretic leg of patients with stroke and was partly explained by poor heel strike or toe-off detection of this leg by inertial sensors leading to higher error of measurement. Another study used Physilog^®^ inertial sensors attached to each shank in patients with diabetes walking on a 50-m tarred pathway and calculated the inter- and intra-session reliability at preferred walking speed [72]. Intra-session relative reliability was comprised between 0.938 and 0.976 for cadence, speed, gait cycle time, stride length, stance phase and double support. However, the intra-session absolute reliability was not reported [72].

### 4.2. Reliability of Dual Task Effects of Gait Parameters in Patients with axSpA

Dual task effects are used to assess the cost of performing another task while walking [51,61]. In the present study, we tested the intra-session reliability of DTE and DTE% in patients with axSpA. Although DTE parameter is frequently used as an outcome in gait studies [13,17,51,73], it is interesting to note that, to the best of our knowledge, its reliability has never been studied in patients with axSpA. Our results showed that the reliability of DTE and DTE% was variable and dependent of the parameter used. The relative reliability was considered as poor to substantial (0.00 < ICC < 0.82) for DTE, and was better for DTE% (0.00 < ICC > 0.85). The absolute reliability of DTE ranged respectively from 0.03 to 4.79 for SEM, and from 0.09 to 13.29 for MDC, and of DTE% from 1.41 to 12.23 for SEM and 3.92 to 33.89 for MDC. If the inter-session reliability of DTE parameters has already been partly assessed in healthy individuals [66,67,74] and pathological populations [13,62,66,68,75,76,77], only few studies have assessed the intra-session reliability of DTE parameters, only in healthy populations [78]. In this study [78], two trials were performed when walking on a 30-m walkway in single- and dual-task (texting) conditions using body worn sensor in 31 healthy young adults (age: 22.5 ± 2.1, 19 females). Gait speed and texting DTE reliability were calculated and relative reliability (ICC) was comprised between 0.76 and 0.91 for gait speed DTE, and 0.03 and 0.22 for texting DTE. Absolute reliability (SEM) was comprised between 3.15 and 4.29 for gait speed DTE and 26.02 and 31.06 for texting DTE. The low relative reliability of DTE could be explained by the addition of measurement errors of single- and dual-task that tend to inflation when combined to obtain DTE [66]. Patients with axSpA we have tested in the present study were asked not to prioritize one task (gait or carry the full cup of water) and may have presented several strategies managing the dual task [67]. Indeed, better inter-session reliability was found when healthy subjects were given instructions about how to prioritize their attention [78]. Taken together, our results suggest that researchers and clinicians should be aware of the poor reliability of DTE among axSpA, particularly for some spatiotemporal gait parameters (e.g., swing, stance, and double support percentages in the present study) and should use speed or stride length which had better DTE reliability.

### 4.3. Number of Trials to Ensure Reliable Measurements

The reliability of gait parameters was substantial to perfect in the single- and the dual-task conditions. Interestingly, we further observed better ICC (i.e., higher), SEM and MDC (i.e., lower) when using gait parameters calculated during the second and the third trials. The reliability was lower when using the first and second trials, the first and third or the three trials. Most of studies on gait used the average of three trials in various populations (e.g., healthy [50,79] or pathological [80]), but the number of trials necessary to obtain reliable measurements has been poorly investigated [28,81]. 

Our results showed that the first trial appears less reliable than the mean of the second and third trials. This is in line with results reported in previous studies [28,81]. For instance, in the study of Brach et al. conducted with 558 healthy older adults, the reliability of gait variability was better when calculated from two 4-m walks than from a single 4-m walk using a Gait Mat II system [81]. These authors explained that by increasing the number of steps in the analysis, it increases the sample size, producing more accurate measure of the real width of the distribution [81]. As well, in the study of Fransen et al. performed on 41 patients with knee osteoarthritis, authors reported greater reliability when using trials 2 and 3 compared to trials 1 and 2 [28]. They also found that the reliability was not increased by increasing the number of trials from two to three, or from two to five. Following these results, these authors advised to allow a familiarization trial at each self-selected walking speed [28]. 

Taken together, these results and ours suggest that the mean of two trials should be used for better reliability of spatiotemporal gait parameters and the first trial should be used, like other studies, as a warming trial (e.g., “The first trial was an exercise to get accustomed to walking protocol and that was not included in further signal analysis” [3]). 

### 4.4. Limitations and Perspectives

This study presents several limitations. First, axSpA being a heterogeneous disease [82], the included population (*n* = 20; disease duration: 11 years) could hence not represent the entire population of axSpA. Moreover, patients presented pain at time at measurements (pain intensity: 3.3) that has been previously reported to influence spatiotemporal gait parameters [83]. Furthermore, only one concurrent and non-verbal task (i.e., a manual task) has been used to assess the reliability of gait performance in dual-task conditions, while gait alterations are dependent of the complexity of the concurrent task [84]. Further, the reliability in the present study was assessed during one session (intra- or within-session reliability), which does not allow the calculation of inter-session reliability. 

Further studies should focus on the assessment of the reliability of gait parameters obtained from foot-worn inertial sensors in other clinical tests that are commonly used in patients with axSpA, like the timed up and go or the 6-min walk test [41,85], and for the assessment of inter-session reliability.

## 5. Conclusions

This study showed that spatiotemporal and symmetry ratio gait parameters measured with foot-worn inertial sensors in patients with axSpA had good reliability in both single- and dual-task walking conditions. This study also provides SEM and MDC values for this population that could be used for future interventions on gait performance. Our findings further suggest that the first trial of each condition could be considered as a warming trial, and the second and the third trials should be averaged to ensure the best reliability of gait measurements in patients with axSpA. Symmetry index and dual task effects parameters reliability was considered as poor, suggesting that clinicians and researchers should be careful when using these outcomes.

## Figures and Tables

**Table 1:** Mean and standard deviation (SD) of spatiotemporal, normalized (N) and symmetry (symmetry index-SI and symmetry ratio-SR) gait parameters obtained in the single-task condition, and ANOVA results.

	Trial 1		Trial 2		Trial 3		Trial 1-2-3		T1-T2	T1-T3	T2-T3
	Mean	SD	Mean	SD	Mean	SD	Mean	SD	*p*-Value	*p*-Value	*p*-Value
**Spatiotemporal parameters**											
Speed	1.24	0.20	1.26	0.20	1.27	0.19	1.26	0.19	0.95	0.86	0.97
Cadence	107.25	9.47	108.20	9.16	108.83	8.70	108.09	8.98	0.94	0.85	0.97
Stride length	1.37	0.17	1.38	0.17	1.38	0.17	1.38	0.17	0.97	0.95	1.00
Swing	38.03	1.69	37.96	1.29	38.15	1.54	38.05	1.49	0.99	0.97	0.92
Stance	61.97	1.69	62.04	1.29	61.85	1.54	61.95	1.49	0.99	0.97	0.92
Double support	23.49	3.51	23.62	2.36	23.35	2.54	23.49	2.81	0.99	0.99	0.95
Load	12.71	3.15	12.69	3.06	13.03	3.25	12.81	3.10	1.00	0.94	0.94
Foot flat	52.54	6.00	52.31	4.79	52.04	5.08	52.30	5.23	0.99	0.95	0.99
Push	34.75	4.00	35.00	2.79	34.94	3.39	34.90	3.37	0.97	0.98	1.00
**Normalized parameters**											
Speed N	0.42	0.06	0.42	0.06	0.43	0.06	0.42	0.06	0.95	0.85	0.97
Cadence N	32.38	2.77	32.58	2.73	32.77	2.56	32.58	2.65	0.97	0.89	0.97
Stride length N	1.52	0.16	1.54	0.16	1.54	0.16	1.54	0.16	0.96	0.93	0.99
**Symmetry index parameters**											
Speed SI	1.60	1.13	2.38	2.81	2.20	2.00	2.06	2.09	0.47	0.63	0.96
Cadence SI	1.01	1.13	1.50	3.03	1.62	2.97	1.38	2.51	0.82	0.73	0.99
Stride length SI	1.46	1.08	1.79	1.48	1.88	1.76	1.71	1.45	0.76	0.64	0.98
Swing SI	3.08	2.07	2.98	2.21	3.28	3.64	3.11	2.69	0.99	0.97	0.94
Stance SI	1.87	1.23	1.80	1.27	1.96	2.06	1.88	1.54	0.99	0.98	0.95
Load SI	10.45	10.12	13.72	9.69	13.08	8.64	12.42	9.45	0.53	0.66	0.98
Foot flat SI	3.66	2.55	5.24	4.25	4.83	4.64	4.58	3.91	0.42	0.62	0.94
Push SI	6.11	4.86	6.42	5.70	5.93	5.75	6.15	5.36	0.98	0.99	0.96
**Symmetry ratio parameters**											
Speed SR	1.01	0.02	1.01	0.04	1.02	0.02	1.01	0.03	1.00	0.37	0.36
Cadence SR	1.00	0.01	1.00	0.03	1.01	0.03	1.00	0.03	0.94	0.36	0.56
Stride length SR	1.01	0.02	1.00	0.02	1.02	0.02	1.01	0.02	0.67	0.59	0.17
Swing SR	1.00	0.04	1.00	0.04	1.02	0.05	1.00	0.04	1.00	0.41	0.40
Stance SR	1.00	0.02	1.00	0.02	0.99	0.03	1.00	0.02	0.99	0.47	0.41
Load SR	1.01	0.15	1.01	0.17	1.03	0.15	1.02	0.16	1.00	0.97	0.96
Foot flat SR	0.99	0.04	0.99	0.06	0.98	0.06	0.98	0.06	0.99	0.83	0.90
Push SR	1.02	0.08	1.02	0.09	1.03	0.08	1.02	0.08	1.00	0.92	0.96

**Table 2:** Mean and standard deviation (SD) of spatiotemporal, normalized (N) and symmetry (symmetry index-SI and symmetry ratio-SR) gait parameters obtained in the dual-task condition, and ANOVA results.

	Trial 1		Trial 2		Trial 3		Trial 1-2-3		T1-T2	T1-T3	T2-T3
	Mean	SD	Mean	SD	Mean	SD	Mean	SD	*p*-Value	*p*-Value	*p*-Value
**Spatiotemporal parameters**											
Speed	1.17	0.21	1.18	0.26	1.17	0.23	1.18	0.23	0.99	1.00	0.98
Cadence	106.91	9.45	106.64	10.80	106.56	11.62	106.71	10.45	1.00	0.99	1.00
Stride length	1.30	0.18	1.30	0.21	1.29	0.17	1.30	0.19	1.00	1.00	0.99
Swing	37.47	1.33	37.48	1.92	37.42	1.46	37.46	1.56	1.00	0.99	0.99
Stance	62.53	1.33	62.52	1.92	62.58	1.46	62.54	1.56	1.00	0.99	0.99
Double support	24.81	2.47	24.13	3.27	24.86	3.07	24.61	2.91	0.76	1.00	0.73
Load	12.79	3.67	12.83	3.74	12.67	3.40	12.76	3.54	1.00	0.99	0.99
Foot flat	52.31	5.36	52.42	6.07	52.73	5.85	52.48	5.66	1.00	0.97	0.99
Push	34.90	3.52	34.76	4.08	34.60	4.21	34.75	3.87	0.99	0.97	0.99
**Normalized parameters**											
Speed N	0.40	0.07	0.40	0.08	0.39	0.07	0.40	0.07	0.99	1.00	0.98
Cadence N	33.00	2.92	32.92	3.33	32.90	3.59	32.94	3.23	1.00	0.99	1.00
Stride length N	1.45	0.17	1.45	0.20	1.45	0.17	1.45	0.18	1.00	1.00	0.99
**Symmetry index parameters**											
Speed SI	1.55	0.96	1.89	2.16	1.30	1.27	1.58	1.53	0.78	0.87	0.48
Cadence SI	0.78	0.45	1.47	2.08	0.65	0.32	0.96	1.27	0.20	0.94	0.11
Stride length SI	1.28	0.79	1.40	1.02	1.33	1.22	1.33	1.00	0.93	0.99	0.98
Swing SI	2.87	2.66	2.38	1.83	4.01	3.72	3.09	2.87	0.85	0.43	0.19
Stance SI	1.72	1.55	1.44	1.13	2.37	2.16	1.84	1.68	0.87	0.44	0.21
Load SI	11.22	8.54	9.44	9.09	15.05	12.21	11.89	10.15	0.84	0.46	0.21
Foot flat SI	5.04	4.78	5.21	3.54	5.64	3.84	5.29	4.04	0.99	0.89	0.95
Push SI	5.53	4.96	6.59	5.11	5.94	4.09	6.01	4.68	0.76	0.96	0.91
**Symmetry ratio parameters**											
Speed SR	1.01	0.01	1.01	0.03	1.01	0.02	1.01	0.02	0.96	0.90	0.98
Cadence SR	1.00	0.01	1.00	0.03	1.00	0.01	1.00	0.02	0.96	0.96	0.85
Stride length SR	1.01	0.01	1.01	0.01	1.01	0.02	1.01	0.01	0.88	0.89	0.63
Swing SR	0.99	0.04	0.99	0.03	1.00	0.06	0.99	0.04	0.98	0.58	0.69
Stance SR	1.01	0.02	1.01	0.02	1.00	0.03	1.01	0.02	0.98	0.61	0.73
Load SR	1.04	0.15	1.01	0.12	1.03	0.18	1.03	0.15	0.87	1.00	0.92
Foot flat SR	0.97	0.06	0.98	0.06	0.97	0.06	0.97	0.06	0.99	1.00	0.98
Push SR	1.02	0.07	1.03	0.08	1.03	0.07	1.03	0.07	1.00	1.00	1.00

**Table 3:** ICC, SEM and MDC values for spatiotemporal, normalized (N) and symmetry (symmetry index-SI and symmetry ratio-SR) gait parameters obtained in the single-task condition between the mean of the first and the second trials (T1-T2), the first and the third trials (T1-T3), the second and the third trials (T2-T3) and the means of the three consecutive trials (T1-T2-T3).

			T1-T2			T1-T3			T2-T3			T1-2-3		
		Parameters	ICC (lb–ub)	SEM(%)	MDC(%)	ICC (lb–ub)	SEM(%)	MDC(%)	ICC (lb–ub)	SEM(%)	MDC(%)	ICC (lb–ub)	SEM(%)	MDC(%)
Almost perfect 0.81 < ICC < 1		**Spatiotemporal parameters**												
		speed	0.96 (0.92–0.98)	2.99	8.29	0.96 (0.87–0.98)	3.24	8.99	0.98 (0.95–0.99)	2.33	6.46	0.97 (0.93–0.98)	2.87	7.95
		cadence	0.95 (0.90–0.98)	1.90	5.28	0.95 (0.88–0.98)	1.89	5.25	0.97 (0.94–0.99)	1.35	3.75	0.96 (0.92–0.98)	1.73	4.79
		stride length	0.98 (0.95–0.99)	1.82	5.04	0.97 (0.94–0.99)	2.02	5.60	0.98 (0.97–0.99)	1.50	4.15	0.98 (0.96–0.99)	1.78	4.94
		DS	0.81 (0.64–0.91)	5.41	14.99	0.59 (0.29–0.79)	8.27	22.91	0.82 (0.64–0.91)	4.43	12.27	0.74 (0.58–0.86)	6.14	17.01
		swing	0.74 (0.52–0.87)	1.98	5.49	0.68 (0.43–0.84)	2.36	6.53	0.85 (0.70–0.93)	1.45	4.01	0.75 (0.60–0.87)	1.96	5.43
		stance	0.74 (0.52–0.87)	1.21	3.36	0.68 (0.43–0.84)	1.45	4.02	0.85 (0.70–0.93)	0.89	2.46	0.75 (0.60–0.87)	1.20	3.33
		LDr	0.93 (0.85–0.97)	6.54	18.13	0.94 (0.87–0.97)	6.24	17.29	0.95 (0.88–0.97)	5.68	15.75	0.94 (0.89–0.97)	6.14	17.01
Substantial 0.61 < ICC < 0.8		FFr	0.90 (0.80–0.95)	3.20	8.87	0.93 (0.85–0.97)	2.83	7.84	0.97 (0.93–0.98)	1.75	4.85	0.93 (0.87–0.96)	2.66	7.36
		Pur	0.86 (0.72–0.93)	3.69	10.24	0.88 (0.76–0.94)	3.68	10.19	0.93 (0.86–0.97)	2.30	6.37	0.89 (0.80–0.94)	3.27	9.08
		**Normalized parameters**												
		speed N	0.96 (0.92–0.98)	2.98	8.25	0.95 (0.86–0.98)	3.21	8.90	0.97 (0.95–0.99)	2.32	6.44	0.96 (0.93–0.98)	2.85	7.90
		cadence N	0.95 (0.89–0.98)	1.90	5.27	0.94 (0.87–0.97)	1.90	5.28	0.97 (0.94–0.99)	1.35	3.75	0.95 (0.92–0.98)	1.73	4.80
		slength N	0.97 (0.93–0.99)	1.81	5.02	0.96 (0.91–0.98)	1.99	5.52	0.98 (0.95–0.99)	1.50	4.15	0.97 (0.94–0.98)	1.77	4.91
Moderate 0.41 < ICC < 0.6		**Symmetry index parameters**												
		speed SI	0.03 (−0.34–0.39)	106.65	295.61	0.08 (−0.28–0.43)	82.51	228.70	0.66 (0.39–0.83)	61.08	169.30	0.32 (0.09–0.56)	83.67	231.93
		cadence SI	0.00 (−0.37–0.37)	181.24	502.38	0.00 (−0.37–0.37)	170.39	472.31	0.68 (0.42–0.84)	107.73	298.61	0.26 (0.03–0.51)	156.66	434.25
		slength SI	0.00 (−0.37–0.37)	79.43	220.16	0.00 (−0.37–0.37)	87.28	241.93	0.25 (−0.12–0.57)	75.81	210.12	0.04 (−0.15–0.3)	83.19	230.60
		swing SI	0.00 (−0.37–0.37)	69.83	193.55	0.15 (−0.23–0.49)	84.89	235.32	0.53 (0.21–0.75)	65.12	180.51	0.26 (0.03–0.51)	74.56	206.66
		stance SI	0.00 (−0.37–0.37)	67.17	186.18	0.13 (−0.25–0.48)	81.53	225.99	0.48 (0.15–0.72)	64.66	179.22	0.23 (0.01–0.49)	71.99	199.54
Fair 0.21 < ICC < 0.4		LDr SI	0.33 (−0.03–0.62)	67.07	185.92	0.56 (0.25–0.77)	52.90	146.63	0.30 (−0.07–0.60)	56.45	156.47	0.40 (0.17–0.63)	59.16	163.99
		FFr SI	0.53 (0.21–0.75)	54.81	151.92	0.55 (0.24–0.76)	59.34	164.48	0.75 (0.53–0.88)	43.68	121.08	0.63 (0.43–0.79)	52.31	144.99
		Pur SI	0.40 (0.05–0.67)	64.37	178.43	0.42 (0.07–0.68)	66.18	183.46	0.72 (0.49–0.86)	48.21	133.62	0.53 (0.31–0.72)	59.91	166.06
		**Symmetry ratio parameters**												
		speed SR	0.22 (−0.16–0.54)	2.56	7.11	0.09 (−0.24–0.42)	2.06	5.72	0.56 (0.25–0.77)	2.07	5.75	0.33 (0.11–0.57)	2.25	6.25
		cadence SR	0.15 (−0.23–0.49)	2.42	6.70	0.12 (−0.22–0.45)	2.53	7.02	0.73 (0.49–0.86)	1.79	4.96	0.40 (0.18–0.63)	2.27	6.30
Slight 0.0 < ICC < 0.2		slength SR	0.13 (−0.25–0.47)	1.82	5.05	0.02 (−0.35–0.38)	1.78	4.95	0.40 (0.06–0.66)	1.73	4.79	0.21 (0.00–0.46)	1.78	4.93
		swing SR	0.36 (0.00–0.64)	3.00	8.32	0.54 (0.23–0.75)	2.97	8.25	0.60 (0.30–0.79)	2.79	7.73	0.52 (0.30–0.71)	2.93	8.11
		stance SR	0.38 (0.02–0.66)	1.74	4.83	0.56 (0.25–0.76)	1.68	4.65	0.55 (0.24–0.76)	1.67	4.63	0.50 (0.29–0.70)	1.70	4.71
		LDr SR	0.62 (0.33–0.8)	9.69	26.86	0.78 (0.58–0.89)	7.01	19.42	0.67 (0.41–0.83)	9.04	25.06	0.69 (0.51–0.83)	8.62	23.90
		FFr SR	0.69 (0.43–0.84)	3.07	8.50	0.67 (0.40–0.83)	3.03	8.41	0.83 (0.67–0.92)	2.60	7.20	0.74 (0.58–0.86)	2.90	8.03
		Pur SR	0.63 (0.34–0.81)	4.92	13.63	0.59 (0.30–0.79)	5.01	13.89	0.80 (0.62–0.90)	3.66	10.15	0.68 (0.50–0.82)	4.55	12.62

**Table 4:** ICC, SEM% and MDC% values for spatiotemporal, normalized (N) and symmetry (symmetry index-SI and symmetry ratio-SR) gait parameters obtained in the dual-task condition between the mean of the first and the second trials (T1-T2), the first and the third trials (T1-T3), the second and the third trials (T2-T3) and the means of the three consecutive trials (T1-T2-T3).

			T1-T2			T1-T3			T2-T3			T1-2-3		
		Parameters	ICC (lb–ub)	SEM(%)	MDC(%)	ICC (lb–ub)	SEM(%)	MDC(%)	ICC (lb–ub)	SEM(%)	MDC(%)	ICC (lb–ub)	SEM(%)	MDC(%)
Almost perfect 0.81 < ICC < 1		**Spatiotemporal parameters**												
		speed	0.96 (0.92–0.98)	3.81	10.55	0.97 (0.95–0.99)	2.93	8.13	0.98 (0.97–0.99)	2.56	7.11	0.97 (0.95–0.99)	3.15	7.95
		cadence	0.96 (0.91–0.98)	1.92	5.31	0.97 (0.93–0.98)	1.75	4.85	0.97 (0.94–0.99)	1.71	4.75	0.97 (0.94–0.98)	1.78	4.79
		stride length	0.97 (0.94–0.99)	2.44	6.76	0.98 (0.96–0.99)	1.84	5.10	0.99 (0.97–0.99)	1.61	4.47	0.98 (0.96–0.99)	2.03	4.94
		DS	0.84 (0.69–0.92)	4.67	12.94	0.81 (0.63–0.91)	4.84	13.41	0.96 (0.91–0.98)	2.72	7.53	0.87 (0.78–0.93)	4.22	17.01
		swing	0.80 (0.61–0.9)	1.96	5.43	0.84 (0.69–0.92)	1.47	4.07	0.91 (0.82–0.96)	1.32	3.65	0.85 (0.75–0.92)	1.61	5.43
		stance	0.80 (0.61–0.9)	1.17	3.25	0.84 (0.69–0.92)	0.88	2.44	0.91 (0.82–0.96)	0.79	2.19	0.85 (0.75–0.92)	0.96	3.33
		LDr	0.98 (0.96–0.99)	3.69	10.24	0.98 (0.95–0.99)	4.21	11.68	0.98 (0.96–0.99)	3.77	10.46	0.98 (0.96–0.99)	3.86	17.01
Substantial 0.61 < ICC < 0.8		FFr	0.97 (0.93–0.98)	1.99	5.52	0.96 (0.93–0.98)	1.98	5.50	0.98 (0.96–0.99)	1.50	4.17	0.97 (0.95–0.99)	1.82	7.36
		Pur	0.94 (0.87–0.97)	2.66	7.36	0.94 (0.87–0.97)	2.78	7.69	0.97 (0.95–0.99)	1.91	5.28	0.95 (0.91–0.98)	2.46	9.08
		**Normalized parameters**												
		speed N	0.96 (0.91–0.98)	3.77	10.45	0.97 (0.94–0.99)	2.94	8.16	0.98 (0.96–0.99)	2.58	7.16	0.97 (0.95–0.99)	3.14	7.90
		cadence N	0.96 (0.91–0.98)	1.92	5.31	0.97 (0.93–0.98)	1.75	4.85	0.97 (0.94–0.99)	1.71	4.75	0.97 (0.94–0.98)	1.78	4.80
		slength N	0.96 (0.93–0.98)	2.38	6.59	0.97 (0.95–0.99)	1.86	5.15	0.98 (0.97–0.99)	1.60	4.43	0.97 (0.95–0.99)	2.00	4.91
Moderate 0.41 < ICC < 0.6		**Symmetry index parameters**												
		speed SI	0.00 (−0.37–0.37)	95.83	265.62	0.05 (−0.33–0.41)	76.06	210.83	0.00 (−0.37–0.37)	111.07	307.86	0.00 (−0.18–0.26)	96.94	231.93
		cadence SI	0.08 (−0.27–0.42)	129.73	359.58	0.26 (−0.11–0.57)	47.70	132.21	0.02 (−0.30–0.37)	142.81	395.84	0.06 (−0.12–0.30)	127.73	434.25
		slength SI	0.57 (0.26–0.78)	44.16	122.39	0.04 (−0.33–0.40)	75.82	210.15	0.08 (−0.29–0.44)	78.02	216.26	0.19 (−0.03–0.45)	67.70	230.60
		swing SI	0.15 (−0.23–0.49)	79.54	220.48	0.48 (0.15–0.72)	67.94	188.31	0.18 (−0.16–0.49)	85.30	236.44	0.30 (0.08–0.55)	77.70	206.66
		stance SI	0.10 (−0.28–0.45)	81.34	225.46	0.43 (0.10–0.69)	69.38	192.30	0.20 (−0.13–0.51)	82.62	229.00	0.28 (0.06–0.53)	77.61	199.54
Fair 0.21 < ICC < 0.4		LDr SI	0.52 (0.19–0.74)	58.55	162.30	0.34 (−0.02–0.62)	65.41	181.31	0.57 (0.25–0.78)	58.65	162.57	0.48 (0.26–0.69)	61.63	163.99
		FFr SI	0.66 (0.38–0.82)	47.74	132.33	0.70 (0.45–0.85)	44.11	122.25	0.79 (0.59–0.89)	31.16	86.38	0.71 (0.54–0.84)	41.00	144.99
		Pur SI	0.69 (0.44–0.84)	46.02	127.55	0.76 (0.54–0.88)	38.80	107.55	0.73 (0.50–0.87)	37.80	104.79	0.72 (0.55–0.85)	41.30	166.06
		**Symmetry ratio parameters**												
		speed SR	0.23 (−0.15–0.55)	1.89	5.25	0.10 (−0.27–0.45)	1.46	4.04	0.26 (−0.12–0.57)	1.92	5.34	0.21 (−0.01–0.47)	1.77	6.25
		cadence SR	0.37 (0.01–0.65)	1.50	4.15	0.51 (0.18–0.74)	0.58	1.60	0.00 (−0.37–0.37)	1.87	5.18	0.20 (−0.02–0.46)	1.43	6.30
Slight 0.0 < ICC < 0.2		slength SR	0.20 (−0.18–0.52)	1.13	3.13	0.00 (−0.37–0.37)	1.45	4.03	0.31 (−0.06–0.61)	1.26	3.50	0.16 (−0.05–0.42)	1.29	4.93
		swing SR	0.13 (−0.25–0.47)	3.05	8.46	0.50 (0.17–0.73)	3.39	9.39	0.40 (0.05–0.67)	3.45	9.57	0.40 (0.18–0.63)	3.25	8.11
		stance SR	0.11 (−0.26–0.46)	1.85	5.12	0.43 (0.09–0.69)	2.06	5.70	0.48 (0.15–0.72)	1.84	5.11	0.40 (0.17–0.63)	1.89	4.71
		LDr SR	0.78 (0.57–0.89)	6.18	17.12	0.68 (0.41–0.84)	9.00	24.95	0.77 (0.56–0.88)	7.28	20.17	0.74 (0.58–0.86)	7.52	23.90
		FFr SR	0.78 (0.58–0.89)	2.85	7.89	0.83 (0.67–0.92)	2.55	7.08	0.87 (0.74–0.94)	2.16	5.98	0.83 (0.71–0.91)	2.52	8.03
		Pur SR	0.67 (0.41–0.83)	4.17	11.57	0.77 (0.56–0.89)	3.30	9.14	0.88 (0.76–0.94)	2.47	6.83	0.77 (0.63–0.88)	3.38	12.62

**Table 5:** Mean and standard deviation (SD) of the dual task effects (DTE) for each trial and for the three trials.

	Trial 1		Trial 2		Trial 3		Trial 1-2-3		T1-T2	T1-T3	T2-T3
	Mean	SD	Mean	SD	Mean	SD	Mean	SD	*p*-Value	*p*-Value	*p*-Value
Speed DTE	0.06	0.10	0.08	0.12	0.09	0.11	0.08	0.11	0.94	0.71	0.89
Cadence DTE	0.34	4.17	1.62	4.04	2.15	5.17	1.35	4.47	0.65	0.42	0.93
Stride length DTE	0.07	0.08	0.07	0.09	0.07	0.08	0.07	0.08	0.99	0.96	0.99
Double support ^a^ DTE	−1.32	2.61	0.72	7.05	−1.51	2.43	−0.71	4.57	0.35	0.99	0.29
Swing DTE	0.56	1.03	0.48	1.32	0.57	1.25	0.54	1.18	0.97	1.00	0.97
Stance ^a^ DTE	−0.56	1.03	−0.48	1.32	−0.57	1.25	−0.54	1.18	0.97	1.00	0.97
Load ^a^ DTE	−0.09	1.64	−0.10	1.61	0.20	1.07	0.00	1.44	1.00	0.82	0.81
Foot flat DTE	0.23	2.54	−0.28	2.96	−0.41	2.94	−0.15	2.78	0.84	0.76	0.99
Push ^a^ DTE	−0.14	2.15	0.38	2.21	0.21	2.23	0.14	2.17	0.74	0.87	0.97

^a^ a negative value is associated to worse performance in the dual-task condition.

**Table 6:** Mean and standard deviation (SD) of the dual task effects percentage (DTE%) for each trial and for the three trials.

	Trial 1		Trial 2		Trial 3		Trial 1-2-3		T1-T2	T1-T3	T2-T3
	Mean	SD	Mean	SD	Mean	SD	Mean	SD	*p*-Value	*p*-Value	*p*-Value
Speed DTE%	5.03	8.43	6.51	9.64	7.69	9.57	6.38	9.12	0.87	0.64	0.92
Cadence DTE%	0.23	3.89	1.60	3.75	2.17	5.13	1.31	4.30	0.59	0.35	0.91
Stride length DTE%	4.96	5.66	5.43	6.64	5.52	5.76	5.30	5.93	0.97	0.95	1.00
Double support ^a^ DTE%	−7.04	13.86	−3.45	10.11	−6.77	10.62	−5.82	11.62	0.62	1.00	0.67
Swing DTE%	1.42	2.69	1.27	3.49	1.45	3.32	1.38	3.12	0.99	1.00	0.98
Stance ^a^ DTE%	−0.94	1.68	−0.77	2.12	−0.93	2.02	−0.88	1.91	0.96	1.00	0.96
Load ^a^ DTE%	−0.64	11.30	−0.46	11.80	1.45	8.39	0.11	10.47	1.00	0.81	0.85
Foot flat DTE%	0.22	4.75	−0.48	5.70	−0.78	5.61	−0.34	5.28	0.91	0.83	0.98
Push ^a^ DTE%	−0.79	6.91	1.23	6.45	0.61	6.56	0.33	6.59	0.61	0.79	0.96

^a^ a negative value is associated to worse performance in the dual-task condition.

**Table 7:** ICC. SEM and MDC for dual task effects parameters (DTE) between the mean of the first and the second trials (T1-T2), the first and the third trials (T1-T3), the second and the third trials (T2-T3) and the means of the three consecutive trials (T1-T2-T3).

		Trials	T1-T2			T1-T3			T2-T3			T1-2-3		
		Parameters	ICC (95% CI)	SEM%	MDC%	ICC (95% CI)	SEM%	MDC%	ICC (95% CI)	SEM%	MDC%	ICC (95% CI)	SEM%	MDC%
Almost perfect 0.81 < ICC < 1		Speed DTE	0.79 (0.55-0.91)	71.78	198.96	0.79 (0.55-0.91)	62.35	172.82	0.82 (0.60-0.92)	57.47	159.31	0.80 (0.64-0.91)	62.94	174.45
		Cadence DTE	0.53 (0.22–0.75)	290.97	806.53	0.61 (0.32–0.80)	240.02	665.31	0.63 (0.35–0.81)	147.37	408.48	0.60 (0.40–0.77)	209.01	579.35
Substantial 0.61 < ICC < 0.8		Stride length DTE	0.78 (0.53 – 0.91)	397.53	150.60	0.80 (0.57 – 0.92)	351.19	130.35	0.81 (0.59 – 0.92)	202.95	132.60	0.80 (0.63 – 0.91)	310.60	137.53
		Double support DTE	0.18 (−0.25–0.56)	200.94	4042.96	0.01 (−0.42–0.44)	185.28	485.97	0.01 (−0.42–0.44)	169.57	485.97	0.19 (-0.06–0.49)	186.45	1597.28
Moderate 0.41 < ICC < 0.6		Swing DTE	0.18 (−0.27–0.57)	54.33	556.97	0.14 (−0.31–0.54)	47.03	513.57	0.51 (0.11 – 0.77)	47.84	470.02	0.28 (0.01 – 0.58)	49.62	516.80
		Stance DTE	0.18 (−0.27–0.57)	200.94	556.97	0.14 (−0.31–0.54)	185.28	513.57	0.51 (0.11 – 0.77)	169.57	470.02	0.28 (0.01 – 0.58)	186.45	516.80
Fair 0.21 < ICC < 0.4		Load DTE	0.65 (0.31 – 0.85)	1458.57	2869.52	0.42 (−0.01–0.72)	175.32	5486.33	0.44 (0.02 – 0.73)	175.32	5446.06	0.52 (0.26 – 0.75)	576.25	69,420.96
Slight 0.0 < ICC < 0.2		Foot flat DTE	0.35 (−0.09 – 0.68)	1035.23	28,642.76	0.43 (0.00 – 0.73)	1979.30	7024.64	0.78 (0.54 – 0.91)	1964.77	1086.47	0.53 (0.27 – 0.76)	25,044.91	3565.81
		Push DTE	0.31 (−0.13 – 0.66)	10,333.41	4432.51	0.28 (−0.17 – 0.63)	2534.27	18,170.05	0.72 (0.42 – 0.88)	391.96	1100.67	0.44 (0.16 – 0.69)	1286.43	3145.73

**Table 8:** ICC. SEM and MDC for dual task effects percent parameters (DTE%) between the mean of the first and second trials (T1-T2). the first and the third trials (T1-T3). the second and the third trials (T2-T3) and the means of the three consecutive trials (T1-T2-T3).

		Trials	T1-T2			T1-T3			T2-T3			T1-2-3		
		Parameters	ICC (95% CI)	SEM%	MDC%	ICC (95% CI)	SEM%	MDC%	ICC (95% CI)	SEM%	MDC%	ICC (95% CI)	SEM%	MDC%
Almost perfect 0.81 < ICC < 1		Speed DTE%	0.75 (0.47–0.89)	78.32	217.09	0.75 (0.48–0.89)	70.58	195.64	0.84 (0.64–0.93)	54.24	150.35	0.78 (0.61–0.90)	66.64	184.71
		Cadence DTE%	0.48 (0.09–0.75)	307.94	853.56	0.50 (0.11–0.77)	274.85	761.86	0.63 (0.28–0.84)	143.21	396.96	0.55 (0.29–0.76)	220.50	611.20
Substantial 0.61 < ICC < 0.8		Stride length DTE%	0.79 (0.55–0.91)	358.55	148.72	0.82 (0.6–0.93)	286.69	125.05	0.85 (0.66–0.94)	201.32	121.19	0.82 (0.67–0.92)	279.50	131.29
		Double support DTE%	0.35 (−0.08–0.68)	195.41	510.94	0.00 (−0.43–0.43)	180.82	490.73	0.59 (0.22–0.81)	166.43	358.26	0.25 (−0.01–0.55)	181.99	479.41
Moderate 0.41 < ICC < 0.6		Swing DTE%	0.14 (−0.31–0.54)	53.65	584.83	0.13 (−0.32–0.53)	45.11	536.88	0.49 (0.09–0.76)	43.72	487.85	0.26 (−0.01–0.56)	47.36	539.75
		Stance DTE%	0.21 (−0.24–0.59)	210.99	541.65	0.14 (−0.31–0.54)	193.69	501.20	0.52 (0.12–0.78)	176.00	461.31	0.30 (0.03–0.59)	194.73	504.46
Fair 0.21 < ICC < 0.4		Load DTE%	0.63 (0.29–0.84)	184.33	3484.78	0.51 (0.11–0.77)	177.04	5058.62	0.46 (0.04–0.74)	129.25	4177.14	0.54 (0.29–0.76)	172.96	18,454.28
Slight 0.0 < ICC < 0.2		Foot flat DTE%	0.35 (−0.09–0.68)	1257.20	9639.47	0.42 (0.00–0.72)	1824.99	4054.84	0.79 (0.54–0.91)	1506.98	1140.65	0.53 (0.27–0.76)	6657.73	2982.74
		Push DTE%	0.75 (0.47–0.89)	3477.62	217.09	0.75 (0.48–0.89)	1462.86	195.64	0.84 (0.64–0.93)	411.51	150.35	0.78 (0.61–0.90)	1076.08	184.71

## Data Availability

The data of the present manuscript can be available on demand to the corresponding author.

## Supplementary Materials

The following are available online at https://www.mdpi.com/1424-8220/20/22/6453/s1, Table S1. LOA, SEM and MDC values for trial 1 and 2, 1 and 3, and 2 and 3 for spatiotemporal, normalized and symmetry gait parameters in single-task condition; Table S2. LOA, SEM and MDC values for trial 1 and 2, 1 and 3, and 2 and 3 for spatiotemporal, normalized and symmetry gait parameters in dual-task condition; Table S3. LOA, SEM and MDC values for trial 1 and 2, 1 and 3, and 2 and 3 for DTE parameters; Table S4. LOA, SEM and MDC values for trial 1 and 2, 1 and 3, and 2 and 3 for DTE% parameters; Figure S1. Bland and Altman plots for speed, cadence and stride length parameters in single task, dual task, and for DTE parameters.

## Abbreviations

axSpA	Axial Spondyloarthritis
DS	double support
DTE	Dual task effects
FFr	Foot flat ratio
FOLOMI	Function Locomotion Measurement Inflammation
ICC	Intraclass Correlation Coefficient
LDr	Load Ratio
MDC	Minimum Detectable Change
N	normalized
PUr	Push ratio
SEM	Standard Error of Measurement
SI	symmetry index
SR	symmetry ratio
