# Supplementary material for: Foot-Worn Inertial Sensors Are Reliable to Assess Spatiotemporal Gait Parameters in Axial Spondyloarthritis under Single and Dual Task Walking in Axial Spondyloarthritis
Source: Sensors (Basel). 2020 Nov 12;20(22):6453. doi: 10.3390/s20226453 (PMC7697708; doi:10.3390/s20226453)

Table S1. LOA, SEM and MDC values for trial 1 and 2, 1 and 3, and 2 and 3 for spatiotemporal, normalized and symmetry parameters in single-task condition.

	Trial 1-2			Trial 1-3			Trial 2-3		
	**LOA (lb - ub)**	**SEM**	**MDC**	**LOA (lb - ub)**	**SEM**	**MDC**	**LOA (lb - ub)**	**SEM**	**MDC**
				Spatiotemporal parameters					
speed	(-0.12 - 0.08)	0.04	0.10	(-0.13 - 0.07)	0.04	0.11	(-0.09 - 0.07)	0.03	0.08
cadence	(-6.39 - 5.11)	2.05	5.68	(-6.58 - 4.06)	2.04	5.66	(-4.65 - 3.41)	1.46	4.06
stride length	(-0.08 - 0.06)	0.02	0.07	(-0.09 - 0.06)	0.03	0.08	(-0.06 - 0.05)	0.02	0.06
DS	(-3.79 - 3.51)	1.27	3.53	(-5.07 - 5.91)	1.94	5.37	(-2.38 - 30.40)	1.04	2.88
swing	(-2.08 - 2.23)	0.75	2.09	(-2.68 - 2.45)	0.90	2.49	(-1.72 - 1.35)	0.55	1.53
stance	(-2.23 - 2.08)	0.75	2.09	(-2.45 - 2.68)	0.90	2.49	(-1.35 - 1.72)	0.55	1.53
LDr	(-2.37 - 2.41)	0.83	2.30	(-2.54 - 10.90)	0.80	2.23	(-2.33 - 1.65)	0.73	2.02
FFr	(-4.57 - 5.04)	1.68	4.65	(-3.63 - 4.64)	1.48	4.10	(-2.30 - 2.84)	0.91	2.53
Pur	(-3.92 - 3.42)	1.29	3.57	(-3.85 - 3.48)	1.28	3.55	(-2.25 - 2.37)	0.80	2.23
				Normalized parameters					
speed norm	(-0.04 - 0.03)	0.01	0.03	(-0.04 - 0.02)	0.01	0.04	(-0.03 - 0.02)	0.01	0.03
cadence norm	(-1.93 - 1.53)	0.62	1.71	(-2.00 - 1.22)	0.62	1.72	(-1.40 - 1.03)	0.44	1.23
slength norm	(-0.09 - 0.06)	0.03	0.08	(-0.10 - 0.06)	0.03	0.08	(-0.07 - 0.06)	0.02	0.06
				Symmetry index parameters					
speed SI	(-6.63 - 5.07)	2.12	5.88	(-4.93 - 3.72)	1.57	4.35	(-3.82 - 4.17)	1.40	3.88
cadence SI	(-7.12 - 6.14)	2.27	6.30	(-7.26 - 6.04)	2.24	6.21	(-4.94 - 4.69)	1.68	4.66
slength SI	(-4.12 - 3.46)	1.29	3.58	(-4.65 - 3.81)	1.46	4.04	(-4.05 - 3.87)	1.39	3.85
swing SI	(-6.12 - 6.31)	2.12	5.87	(-7.88 - 7.48)	2.70	7.48	(-6.11 - 5.51)	2.04	5.65
stance SI	(-3.51 - 3.64)	1.23	3.42	(-4.54 - 4.36)	1.56	4.33	(-3.63 - 3.31)	1.22	3.37
LDr SI	(-25.59 - 19.06)	8.11	22.47	(-19.73 - 14.48)	6.22	17.25	(-20.92 - 22.20)	7.56	20.97
FFr SI	(-7.95 - 4.80)	2.44	6.76	(-8.05 - 5.7)	2.52	6.99	(-5.86 - 6.67)	2.20	6.10
Pur SI	(-11.83 - 11.2)	4.03	11.18	(-11.21 - 11.57)	3.98	11.04	(-8.00 - 8.98)	2.98	8.25
				Symmetry ratio parameters					
speed SR	(-0.07 - 0.07)	0.03	0.07	(-0.07 - 0.04)	0.02	0.06	(-0.07 - 0.04)	0.02	0.06
cadence SR	(-0.07 - 0.07)	0.02	0.07	(-0.08 - 0.06)	0.03	0.07	(-0.06 - 0.04)	0.02	0.05
slength SR	(-0.05 - 0.06)	0.02	0.05	(-0.06 - 0.04)	0.02	0.05	(-0.06 - 0.03)	0.02	0.05
swing SR	(-0.09 - 0.09)	0.03	0.08	(-0.10 - 0.06)	0.03	0.08	(-0.09 - 0.06)	0.03	0.08
stance SR	(-0.05 - 0.05)	0.02	0.05	(-0.04 - 0.05)	0.02	0.05	(-0.03 - 0.05)	0.02	0.05
LDr SR	(-0.28 - 0.28)	0.10	0.27	(-0.22 - 0.19)	0.07	0.20	(-0.28 - 0.25)	0.09	0.26
FFr SR	(-0.08 - 0.09)	0.03	0.08	(-0.07 - 0.09)	0.03	0.08	(-0.06 - 0.08)	0.03	0.07
Pur SR	(-0.15 - 0.14)	0.05	0.14	(-0.16 - 0.14)	0.05	0.14	(-0.11 - 0.10)	0.04	0.10

Abbreviations: norm = normalized, slength = stride length, DS = double support, LDr = Load Ratio, FFr= Foot flat ratio, Pur = Push ratio, SI = symmetry index, SR = symmetry ratio

Table S2. LOA, SEM and MDC values for trial 1 and 2, 1 and 3, and 2 and 3 for spatiotemporal, normalized and symmetry parameters in dual-task condition.

	**Trial 1-2**			**Trial 1-3**			**Trial 2-3**		
	**LOA (lb - ub)**	**SEM**	**MDC**	**LOA (lb - ub)**	**SEM**	**MDC**	**LOA (lb - ub)**	**SEM**	**MDC**
				Spatiotemporal parameters					
speed	(-0.14 - 0.12)	0.04	0.12	(-0.11 - 0.09)	0.03	0.10	(-0.09 - 0.08)	0.03	0.08
cadence	(-5.15 - 6.32)	2.05	5.67	(-5.04 - 5.57)	1.87	5.18	(-5.55 - 4.67)	1.83	5.06
stride length	(-0.10 - 0.08)	0.03	0.09	(-0.08 - 0.06)	0.02	0.07	(-0.07 - 0.05)	0.02	0.06
DS	(-2.70 - 3.63)	1.14	3.17	(-3.3 - 3.63)	1.20	3.33	(-2.13 - 1.81)	0.67	1.85
swing	(-2.13 - 2.07)	0.73	2.03	(-1.65 - 1.54)	0.55	1.52	(-1.57 - 1.29)	0.49	1.37
stance	(-2.07 - 2.13)	0.73	2.03	(-1.54 - 1.65)	0.55	1.52	(-1.29 - 1.57)	0.49	1.37
LDr	(-1.35 - 1.35)	0.47	1.31	(-1.59 - 1.49)	0.54	1.49	(-1.43 - 10.30)	0.48	1.33
FFr	(-3.22 - 2.63)	1.04	2.89	(-3.14 - 2.83)	1.04	2.89	(-2.00 - 2.45)	0.79	2.19
Pur	(-2.29 - 2.88)	0.93	2.56	(-2.53 - 2.93)	0.96	2.67	(-2.02 - 10.70)	0.66	1.83
				Normalized parameters					
speed norm	(-0.05 - 0.04)	0.01	0.04	(-0.04 - 0.03)	0.01	0.03	(-0.03 - 0.03)	0.01	0.03
cadence norm	(-1.59 - 1.95)	0.63	1.75	(-1.55 - 1.72)	0.58	1.60	(-1.71 - 1.44)	0.56	1.56
slength norm	(-0.11 - 0.08)	0.03	0.10	(-0.09 - 0.06)	0.03	0.07	(-0.08 - 0.06)	0.02	0.06
				Symmetry index parameters					
speed SI	(-5.06 - 4.42)	1.64	4.55	(-2.72 - 3.35)	1.09	3.01	(-4.41 - 5.64)	1.77	4.91
cadence SI	(-4.66 - 3.35)	1.45	4.02	(-0.81 - 1.09)	0.34	0.95	(-3.29 - 5.06)	1.51	4.19
slength SI	(-1.81 - 1.53)	0.59	1.63	(-2.87 - 2.81)	0.99	2.74	(-3.07 - 3.07)	1.06	2.95
swing SI	(-5.53 - 6.34)	2.09	5.81	(-7.39 - 5.39)	2.33	6.46	(-8.95 - 5.93)	2.73	7.56
stance SI	(-3.43 - 3.87)	1.29	3.57	(-4.46 - 3.31)	1.41	3.91	(-5.15 - 3.44)	1.57	4.36
LDr SI	(-16.02 - 18.32)	6.06	16.80	(-27 - 20.23)	8.56	23.73	(-23.25 - 13.16)	7.18	19.91
FFr SI	(-6.98 - 7.08)	2.45	6.78	(-7.12 - 6.28)	2.35	6.52	(-5.31 - 4.17)	1.69	4.69
Pur SI	(-8.92 - 6.29)	2.78	7.71	(-6.65 - 6.04)	2.22	6.16	(-5.75 - 7.47)	2.37	6.57
				Symmetry ratio parameters					
speed SR	(-0.05 - 0.06)	0.02	0.05	(-0.04 - 0.05)	0.01	0.04	(-0.06 - 0.06)	0.02	0.05
cadence SR	(-0.04 - 0.04)	0.01	0.04	(-0.02 - 0.02)	0.01	0.02	(-0.06 - 0.05)	0.02	0.05
slength SR	(-0.03 - 0.03)	0.01	0.03	(-0.04 - 0.04)	0.01	0.04	(-0.03 - 0.04)	0.01	0.04
swing SR	(-0.09 - 0.08)	0.03	0.08	(-0.11 - 0.08)	0.03	0.09	(-0.11 - 0.09)	0.03	0.10
stance SR	(-0.05 - 0.05)	0.02	0.05	(-0.05 - 0.07)	0.02	0.06	(-0.05 - 0.06)	0.02	0.05
LDr SR	(-0.13 - 0.21)	0.06	0.18	(-0.26 - 0.28)	0.09	0.26	(-0.24 - 0.18)	0.07	0.21
FFr SR	(-0.08 - 0.07)	0.03	0.08	(-0.07 - 0.07)	0.02	0.07	(-0.05 - 0.06)	0.02	0.06
Pur SR	(-0.13 - 0.12)	0.04	0.12	(-0.10 - 0.10)	0.03	0.09	(-0.07 - 0.08)	0.03	0.07

Abbreviations: norm = normalized, slength = stride length, DS = double support, LDr = Load Ratio, FFr= Foot flat ratio, Pur = Push ratio, SI = symmetry index, SR = symmetry ratio

Table S3. LOA, SEM and MDC values for trial 1 and 2, 1 and 3, and 2 and 3 for DTE parameters

	Trial 1-2			Trial 1-3			Trial 2-3		
	**LOA (lb - ub)**	**SEM**	**MDC**	**LOA (lb - ub)**	**SEM**	**MDC**	**LOA (lb - ub)**	**SEM**	**MDC**
speed	(-0.15 - 0.13)	0.05	0.14	(-0.16 - 0.11)	0.05	0.14	(-0.15 - 0.13)	0.05	0.14
cadence	(-9.02 - 6.38)	2.81	7.80	(-9.38 - 6.35)	2.94	8.14	(-8.27 - 7.67)	2.78	7.71
stride length	(-0.11 - 0.11)	0.04	0.11	(-0.10 - 0.09)	0.03	0.09	(-0.10 - 0.10)	0.04	0.10
DS	(-15.36 - 11.22)	4.79	13.29	(-6.85 - 7.35)	2.48	6.86	(-6.85 - 7.35)	2.48	6.86
swing	(-2.92 - 3.12)	1.05	2.91	(-3.04 – 3.00)	1.05	2.91	(-2.63 - 2.46)	0.89	2.46
stance	(-3.12 - 2.92)	1.05	2.91	(-3.00 - 3.04)	1.05	2.91	(-2.46 - 2.63)	0.89	2.46
LDr	(-2.75 - 2.66)	0.94	2.61	(-3.25 - 2.66)	1.05	2.90	(-3.07 - 2.69)	1.01	2.80
FFr	(-5.58 - 6.81)	2.20	6.09	(-5.17 - 6.39)	2.05	5.69	(-3.93 - 3.82)	1.36	3.76
Pur	(-5.61 - 4.47)	1.80	4.98	(-5.59 - 4.95)	1.85	5.11	(-3.07 - 3.55)	1.17	3.24

Abbreviations: slength = stride length, DS = double support, LDr = Load Ratio, FFr= Foot flat ratio, Pur = Push ratio

Table S4. LOA, SEM and MDC values for trial 1 and 2, 1 and 3, and 2 and 3 for DTE% parameters

	Trial 1-2			Trial 1-3			Trial 2-3		
	**LOA (lb - ub)**	**SEM**	**MDC**	**LOA (lb - ub)**	**SEM**	**MDC**	**LOA (lb - ub)**	**SEM**	**MDC**
speed	(-14.00 - 11.14)	4.50	12.48	(-14.27 - 9.58)	4.46	12.37	(-11.65 - 10.16)	3.85	10.67
cadence	(-8.91 - 6.10)	2.76	7.66	(-10.31 - 6.99)	3.23	8.95	(-8.07 - 7.35)	2.69	7.47
stride length	(-8.28 - 7.62)	2.78	7.72	(-7.15 - 6.22)	2.36	6.54	(-6.69 - 6.96)	2.39	6.64
DS	(-31.64 - 23.01)	9.84	27.28	(-35.23 - 35.48)	12.23	33.89	(-14.22 - 21.69)	6.66	18.47
swing	(-7.98 - 8.35)	2.84	7.86	(-8.08 - 7.93)	2.77	7.69	(-7.04 - 6.69)	2.39	6.62
stance	(-5.00 - 4.61)	1.67	4.64	(-4.84 - 4.90)	1.69	4.69	(-3.9 - 4.21)	1.41	3.92
LDr	(-20.31 - 19.16)	6.89	19.09	(-21.60 - 17.3)	6.93	19.21	(-22.55 - 20.26)	7.48	20.73
FFr	(-10.99 - 12.73)	4.17	11.57	(-10.13 – 12.00)	3.90	10.82	(-7.41 - 7.35)	2.58	7.16
Pur	(-18.03 - 13.65)	5.69	15.79	(-17.91 - 15.25)	5.83	16.17	(-9.21 - 10.88)	3.56	9.85

Abbreviations: slength = stride length, DS = double support, LDr = Load Ratio, FFr= Foot flat ratio, Pur = Push ratio

Figure S1. Bland and Altman plots for speed, cadence and stride length parameters in single task (ST), dual task (DT), and for dual task effects (DTE and DTE%) parameters

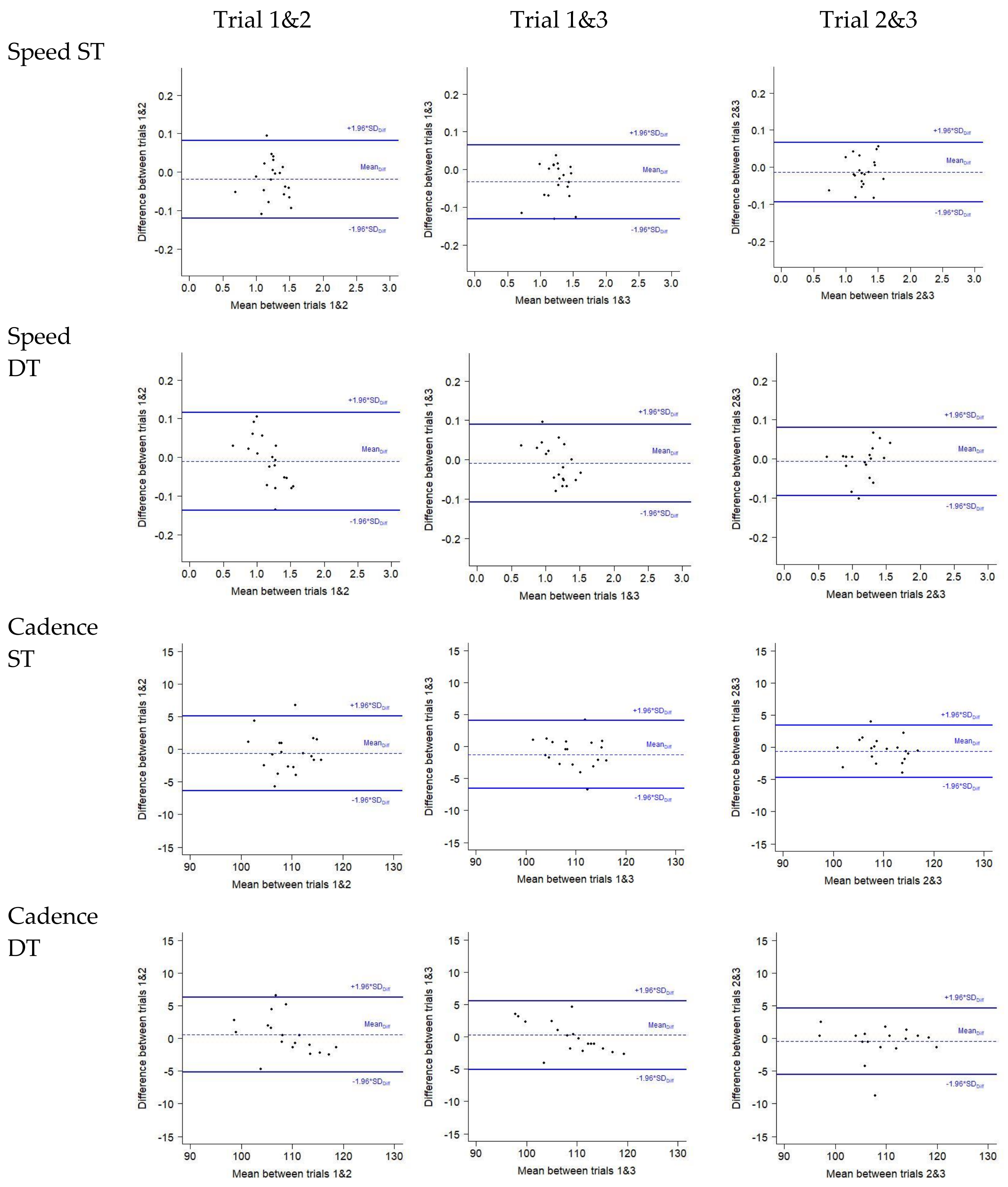

Stride length ST

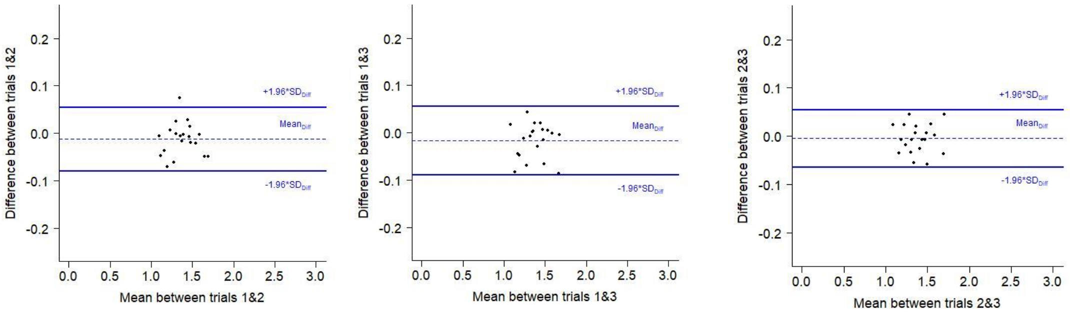

Stride length DT

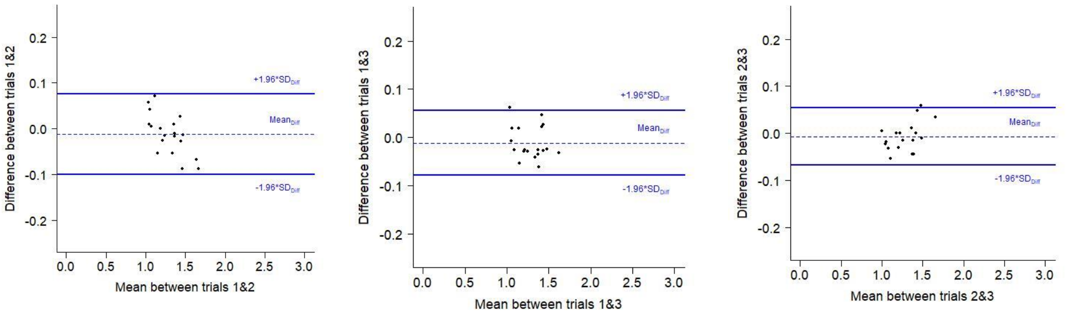

Speed DTE

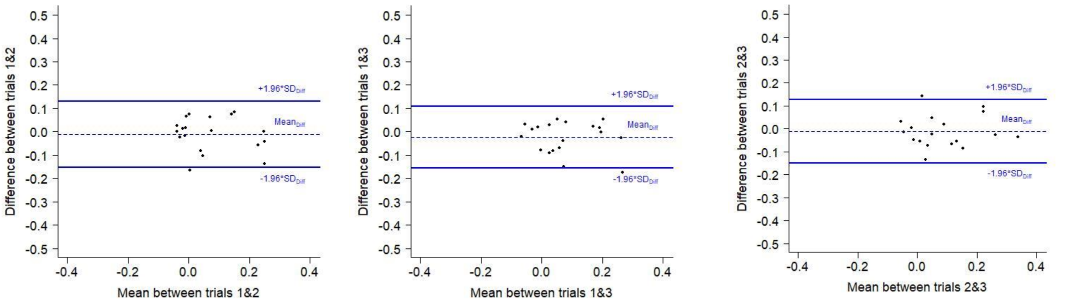

Cadence DTE

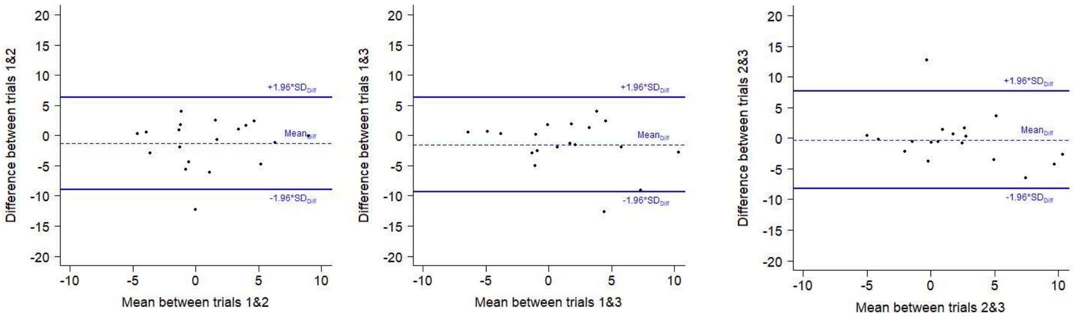

Stride length DTE

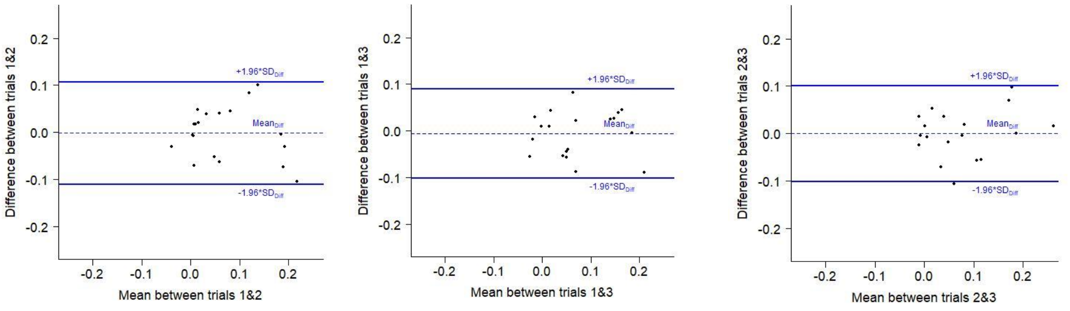

Speed
DTE%

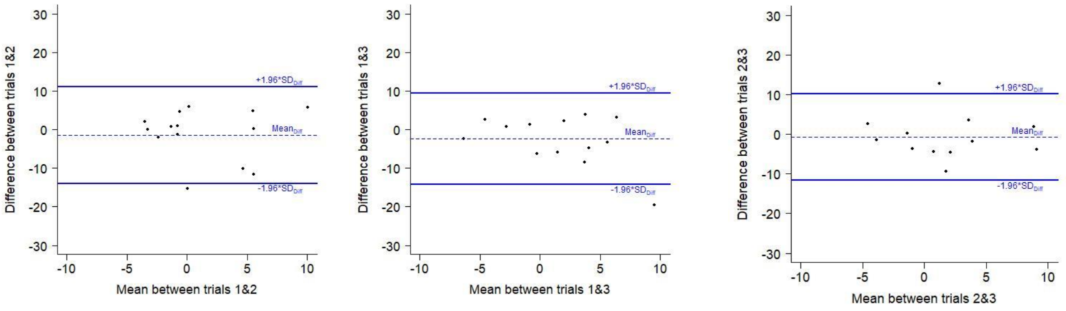

Cadence
DTE%

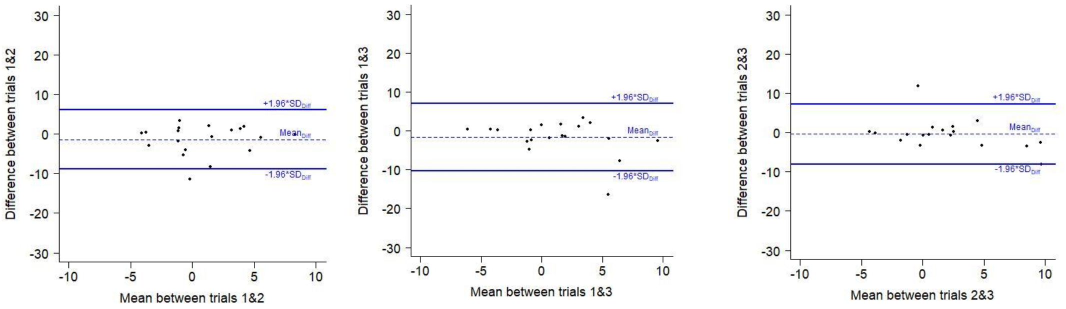

Stride
length
DTE%

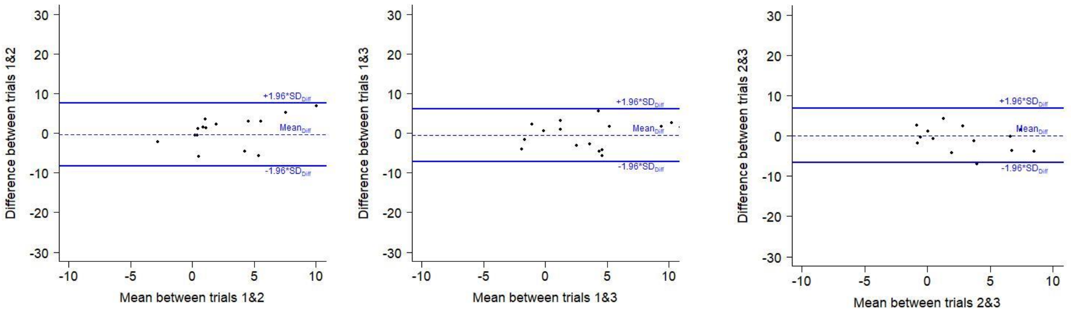

Supplement: Supplementary file 1 [file sensors-20-06453-s001.pdf]
